# Elucidating mechanism of optical cavities in superconducting strip single photon detectors using transmission line and impedance models

**DOI:** 10.1038/s41598-026-52711-4

**Published:** 2026-05-18

**Authors:** Hiroki Kutsuma, Taro Yamashita

**Affiliations:** Graduate School of Engineering, Tohoku University, Sendai, Miyagi 980-8579 Japan

**Keywords:** Engineering, Optics and photonics, Physics

## Abstract

We clarified the physical mechanism of superconducting strip single photon detectors (SSPDs) with optical cavities by using transmission line and impedance models. By introducing the transmission line model, we derived the analytical formulae for the absorptance of SSPDs with optical cavities. We compared the absorptance obtained from the analytical formulae for SSPDs with single-side, double-side, and dielectric multi-layer optical cavities against the results of numerical simulations. The comparison showed that the results were nearly identical. By introducing the impedance model, it was clearly shown that the SSPDs with optical cavities achieved the maximum absorptance when their input impedance of the SSPDs with optical cavities matched the impedance of the input medium. The design concepts proposed in this study are applicable to other superconducting detectors, such as microwave kinetic inductance detectors and transition-edge sensors.

## Introduction

Superconducting strip single photon detectors (SSPDs)^1^ are widely used in quantum computing^2^, quantum key distribution^3^, and quantum optics experiments^4,5^ because they offer high detection efficiencies^6–12^, low timing jitters^13–15^, low dark count rates^16–19^, and high count rates^20–23^. Among these properties, detection efficiency is the most critical parameter for photon detectors. The system detection efficiency (SDE) is defined as $\textrm{SDE} = P_\textrm{pulse}\times P_\textrm{couple}\times P_\textrm{abs}\times (1 - P_\textrm{loss})$, where $P_\textrm{pulse}$ is the pulse generation efficiency, $P_\textrm{couple}$ is the coupling efficiency, $P_\textrm{abs}$ is the absorptance, and $P_\textrm{loss}$ denotes other losses. With various advancements^6,24–32^, SSPDs have achieved SDEs approaching unity at telecommunication wavelengths^8–10,12^, offering a significantly higher detection efficiency than that of semiconductor detectors, such as avalanche photodiodes (APDs)^33^ and frequency upconversion detectors^34^.

The introduction of optical cavities^25^ has played a key role in significantly enhancing the absorptance $P_\textrm{abs}$. Optical cavities store incident photons and enable efficient interaction with the superconducting wire. The geometry of the optical cavities has been optimized through numerical simulations^7,35,36^, such as rigorous coupled-wave analysis (RCWA)^37^ or finite element method (FEM)^38^. The simulation results reveal that the maximum absorptance strongly depends on the thickness and line-to-space ratio of the superconducting wire^36^. Furthermore, it was found that in optical cavities with metallic mirrors, the thickness of the dielectric layer for achieving the maximum absorptance does not necessarily match the quarter-wave thickness of the incident photon wavelength scaled by its refractive index. In^36^, the absorptance of the SSPDs with optical cavities was evaluated for various filling factors and their absorptance was simulated using a commercial FEM solver. The experimentally measured absorptance tended to follow that predicted by the simulation. This indicates that the simulations provide insight into the absorptance of SSPDs with optical cavities. However, the underlying physical mechanism of SSPDs with optical cavities, by which they achieve the desired performance in simulations, remains unclear. To address this gap, several analytical approaches have been proposed. In^39^, the impedance model was introduced to analytically calculate the absorptance as a function of the thickness of a superconducting wire sandwiched between two dielectric layers with infinite thicknesses. The maximum absorptance depends only on the refractive indices of the two dielectric layers, whereas the required thickness of the superconducting wire to achieve the maximum absorptance depends only on the refractive indices of the two dielectric layers, the superconducting material, slit material, and filling factor. More recently, the transmission line model, which is commonly used in the design of microwave circuits, was applied to calculate the absorptance of the optical cavities in microwave kinetic inductance detectors (MKIDs)^40^. However, these approaches are not applicable to the calculation of SSPDs with optical cavities, and do not provide the physical mechanism for achieving the maximum absorptance of such SSPDs.

In this study, we applied the transmission line and impedance models^41^ to the design of SSPDs with optical cavities to provide design guidelines with physical considerations. We propose a design methodology for SSPDs with optical cavities and derive the analytical formulae for the thicknesses of the superconducting wire and dielectric layer required to achieve the maximum absorptance. Finally, we clarify the physical mechanism underlying the maximum absorptance.

## Transmission line model for SSPDs

To apply the transmission line model to the design of SSPDs with optical cavities, we impose the following conditions: The wavelength of the incident photon is much longer than the wire width, such as in superconducting nanostrip single photon detectors (SNSPDs), or much shorter than the wire width, such as in superconducting wide strip single photon detectors (SWSPDs)^42^ (Condition I). For the calculation of SNSPDs, the geometry is assumed to be a meander structure^24^, and the polarization of the incident photon is aligned parallel to the wire axis (Condition II). The loss in the dielectric layer is negligible (Condition III). The relative permeabilities of both the metal and dielectric layers are equal to 1 (Condition IV). The imaginary part of the refractive index of the mirror is much larger than its real part, as in the case with commonly used materials such as silver and gold in the optical cavities^6,10,32,36^ (Condition V). The thickness of the mirror is sufficient to prevent the penetration of the incident photons (Condition VI). The superconducting wire is much thinner than the wavelength of the incident photon (Condition VII). In this paper, the impedance, wavelength, and wave number of vacuum are denoted as $\eta _0$, $\lambda _0$, and $k_0$, respectively. We also define the complex relative permittivity $\varepsilon _\textrm{M}$ (complex refractive index $n_\textrm{M}$) of a metal as $\varepsilon _\textrm{M} = \varepsilon _\textrm{Mr} - i\varepsilon _\textrm{Mi}$ ($n_\textrm{M} = n_\textrm{Mr} - in_\textrm{Mi}$), where $\varepsilon _\textrm{Mr}$ ($n_\textrm{Mr}$) and $\varepsilon _\textrm{Mi}$ ($n_\textrm{Mi}$) represent the real and imaginary parts of the complex relative permittivity (complex refractive index) of the metal. Note that $\varepsilon _\textrm{Mr}$, $\varepsilon _\textrm{Mi}$, $n_\textrm{Mr}$, and $n_\textrm{Mi}$ are positive values and the complex relative permittivity is related to the refractive index given by $n_\textrm{M} = \sqrt{\varepsilon _\textrm{M}}$.

The structure of SSPDs with optical cavities is regarded as a stacked arrangement consisting of the superconducting wire layer, dielectric layer, and metallic mirror layer, sandwiched between the input and output media. The *F* matrix of the layer $\textrm{x}$ is given by the following formula^40,41^:

1 $$\begin{aligned} F_\textrm{x} = \begin{pmatrix} F_{\textrm{x}, 11}& F_{\textrm{x}, 12}\\ F_{\textrm{x}, 21}& F_{\textrm{x}, 22} \end{pmatrix} = \begin{pmatrix} \cosh (\gamma _\textrm{x}d_\textrm{x})& \eta _\textrm{x}\sinh (\gamma _\textrm{x}d_\textrm{x})\\ \frac{1}{\eta _\textrm{x}}\sinh (\gamma _\textrm{x}d_\textrm{x})& \cosh (\gamma _\textrm{x}d_\textrm{x}) \end{pmatrix}, \end{aligned}$$

where $d_\textrm{x}$, $\eta _\textrm{x}$, and $\gamma _\textrm{x}$ are the thickness, characteristic impedance, and propagation constant of the layer x, respectively. Here, the subscript x may represent the wire layer  ($\textrm{w}$), dielectric layer ($\textrm{c}$), or metallic mirror layer ($\textrm{m}$). In Condition IV, $\eta _\textrm{x}$ and $\gamma _\textrm{x}$ are defined as $\eta _\textrm{x} = \eta _0/n_\textrm{x}$ and $\gamma _\textrm{x} = ik_0n_\textrm{x}$, where $n_\textrm{x}$ is the complex refractive index of the layer x. Similar to the transmission line of microwave circuits, the *F* matrix of the total structure $F_\textrm{t}$ is given by the product of each matrix from the input to output media. The absorptance of SSPDs with optical cavities is given by

2 $$\begin{aligned} A = 1 - \bar{r}r - \bar{t}t, \end{aligned}$$

where *r* and *t* are the reflection and transmission coefficients, respectively, and $\bar{r}$ and $\bar{t}$ are their complex conjugates. In^43^, *r* and *t* are expressed as

3 $$\begin{aligned} r = \frac{F_\textrm{t, 11}\eta _\textrm{o} + F_\textrm{t, 12} - F_\textrm{t, 21}\bar{\eta _\textrm{i}}\eta _\textrm{o} - F_\textrm{t, 22}\bar{\eta _\textrm{i}}}{F_\textrm{t, 11}\eta _\textrm{o} + F_\textrm{t, 12} + F_\textrm{t, 21}\eta _\textrm{i}\eta _\textrm{o} + F_\textrm{t, 22}\eta _\textrm{i}} \end{aligned}$$

and

4 $$\begin{aligned} t = \frac{2\sqrt{\textrm{Re}(\eta _\textrm{i})\textrm{Re}(\eta _\textrm{o})}}{F_\textrm{t, 11}\eta _\textrm{o} + F_\textrm{t, 12} + F_\textrm{t, 21}\eta _\textrm{i}\eta _\textrm{o} + F_\textrm{t,22}\eta _\textrm{i}}, \end{aligned}$$

where $\eta _\textrm{i}$ and $\eta _\textrm{o}$ are the impedances of the input and output media and $\bar{\eta _\textrm{i}}$ represents the complex conjugate of ${\eta _\textrm{i}}$. From Condition VI, the second term of the right-hand side ($\bar{t}t$) in Eq. (2) is negligible. By satisfying Condition I and Condition II, the complex relative permittivity of the wire layer $\varepsilon _\textrm{w}$, which is defined as the layer included in the superconducting wire and slit dielectric, is given by^39,44^

5 $$\begin{aligned} \varepsilon _\textrm{w} = \varepsilon _\textrm{metal}f + \varepsilon _\textrm{slit}(1 - f), \end{aligned}$$

where $\varepsilon _\textrm{metal}$ and $\varepsilon _\textrm{slit}$ are the complex relative permittivities of the metal and slit dielectric, respectively, and *f* is the filling factor, which is the ratio of the line width to the sum of line and slit widths. Because the superconducting wire is much thinner than the wavelength of the incident photon (Condition VII), the *F* matrix of the wire layer, $F_\textrm{w}$, is approximated by

6 $$\begin{aligned} F_\textrm{w} \approx \begin{pmatrix} 1& \eta _\textrm{w}\gamma _\textrm{w}d_\textrm{w}\\ \gamma _\textrm{w}d_\textrm{w}/\eta _\textrm{w}& 1 \end{pmatrix}, \end{aligned}$$

where $\gamma _\textrm{w}$, $\eta _\textrm{w}$, and $d_\textrm{w}$ are the propagation constant, characteristic impedance, and thickness of the wire layer, respectvely.

Because we have set the dielectric loss in the dielectric layer as negligible (Condition III) and its thickness is commonly considered as nearly one-quarter of the incident photon wavelength scaled by its refractive index, the *F* matrix of the dielectric layer $F_\textrm{c}$ is approximated by

7 $$\begin{aligned} F_\textrm{c} \approx \begin{pmatrix} -\Delta \phi _\textrm{c}& i\eta _\textrm{c}\\ i/\eta _\textrm{c}& -\Delta \phi _\textrm{c} \end{pmatrix}. \end{aligned}$$

**Fig. 1:**
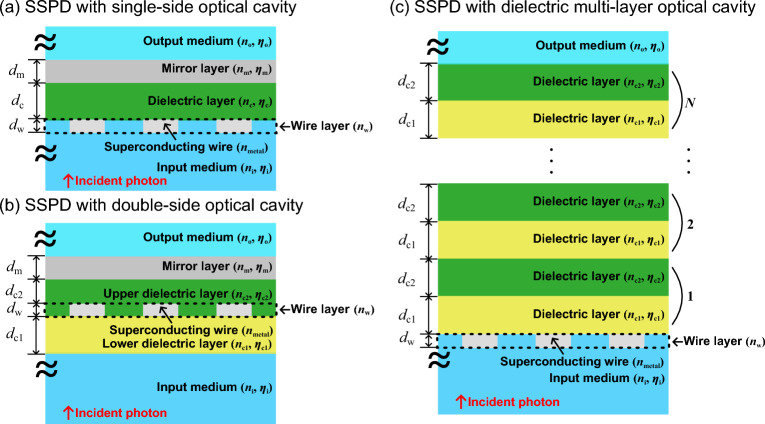
Schematics of SSPDs with optical cavities. (**a**) SSPD with single-side optical cavity. (**b**) SSPD with double-side optical cavity. (**c**) SSPD with dielectric multi-layer optical cavity. The two dielectric layers with different refractive indices are stacked in *N* periods.

$\Delta \phi _\textrm{c}$ is defined as $\phi _\textrm{c} = \pi /2 + \Delta \phi _\textrm{c}$, where $\phi _\textrm{c} = k_0n_\textrm{c}d_\textrm{c}$, and $n_\textrm{c}$ and $d_\textrm{c}$ are the refractive index and thickness of the dielectric layer, respectively. Note that $\Delta \phi _\textrm{c}$ is equal to 0 when the thickness of the dielectric layer is one-quarter of the incident photon wavelength scaled by its refractive index. In Condition VI, the output medium is replaced by a metallic mirror, i.e., the output medium is treated as a metallic mirror with infinite thickness. Therefore, the characteristic impedance of the output medium, $\eta _\textrm{o}$, is replaced with $\eta _\textrm{o}\approx i\textrm{Im}(\eta _\textrm{m})$. This approximation is based on the fact that the imaginary part of the refractive index of the metallic mirror is much larger than its real part (Condition V). Using the above equations and approximations, we apply the transmission line model to SSPDs with single-side optical cavities (Fig. 1a), double-side optical cavities (Fig. 1b), and dielectric multi-layer optical cavities (Fig. 1c). All these configurations are reported to achieve high system detection efficiencies^6–12,32,36^.

### Single-side optical cavity

Here, we apply the transmission line model to SSPDs with single-side optical cavities, which consist of a dielectric layer with a metallic mirror placed on the superconducting wire, as shown in Fig. 1a. First, to derive the thickness of the wire layer that achieve the maximum absorptance, we simplify the material of the mirror as a perfect electrical conductor (PEC), i.e., $\eta _\textrm{m} = 0$, and the thickness of the dielectric layer as one-quarter of the incident photon wavelength scaled by its refractive index, i.e., $\Delta \phi _\textrm{c} = 0$. Based on the approximations given in Eqs. (6) and  (7), the total *F* matrix of the SSPDs with single-side optical cavities, $F_\textrm{SSC}$, is approximated by

8 $$\begin{aligned} F_\textrm{SSC} = F_\textrm{w}\times F_\textrm{c} = \begin{pmatrix} i\eta _\textrm{w}\gamma _\textrm{w}d_\textrm{w}/\eta _\textrm{c}& i\eta _\textrm{c}\\ i/\eta _\textrm{c}& i\eta _\textrm{c}\gamma _\textrm{w}d_\textrm{w}/\eta _\textrm{w} \end{pmatrix}. \end{aligned}$$

By substituting Eq. (8) into Eqs. (2) and  (3), the absorptance of the SSPDs with single-side optical cavities, $A_\textrm{SSC}$, is given by

9 $$\begin{aligned} A_\textrm{SSC} = \frac{-4k_0\textrm{Im}(\varepsilon _\textrm{w})n_\textrm{i}d_\textrm{w}}{(n_\textrm{i} - k_0\textrm{Im}(\varepsilon _\textrm{w})d_\textrm{w})^2 +~(k_0\textrm{Re}(\varepsilon _\textrm{w})d_\textrm{w})^2}, \end{aligned}$$

where $n_\textrm{i}$ is the refractive index of the input medium. An important point to note is that the absorptance is characterized only by the refractive index of the input medium, complex relative permittivity, and thickness of the wire layer. The maximum absorptance $A_\textrm{SSC}^\textrm{max}$ and thickness of the wire layer at the maximum absorptance, $d^\textrm{max}_\textrm{w, SSC}$, satisfy $\textrm{d} A_\textrm{SSC}/\textrm{d}d_\textrm{w} = 0$, resulting in

10 $$\begin{aligned} d^\textrm{max}_\textrm{w, SSC} = \frac{n_\textrm{i}}{k_0|\varepsilon _\textrm{w}|} \end{aligned}$$

and

11 $$\begin{aligned} A_\textrm{SSC}^\textrm{max} = \frac{2\textrm{Im}(\varepsilon _\textrm{w})}{\textrm{Im}(\varepsilon _\textrm{w}) - |\varepsilon _\textrm{w}|}. \end{aligned}$$

As a result, we found that the required thickness of the wire layer to achieve the maximum absorptance was characterized by the refractive index of the input medium and complex relative permittivity of the wire layer, whereas $A_\textrm{SSC}^\textrm{max}$ is characterized by the complex relative permittivity of the wire layer. The results indicate that the thickness of the wire layer at the maximum absorptance depends on the filling factor, because the relative permittivity of the wire layer depends on the filling factor, as shown in Eq. (5).

Next, we derive the thickness of the dielectric layer at the maximum absorptance. In this calculation, we fix the thickness of the wire layer at the maximum absorptance determined by Eq. (10). Here, we assume the metallic mirror, i.e., $\eta _\textrm{m} \ne 0$. In this condition, the *F* matrix is approximated by

12 $$\begin{aligned} F'_\textrm{SSC} = \begin{pmatrix} -\Delta \phi _\textrm{c} + i\eta _\textrm{w}\gamma _\textrm{w}d^\textrm{max}_\textrm{w, SSC}/\eta _\textrm{c}& i\eta _\textrm{c}\\ i/\eta _\textrm{c}& -\Delta \phi _\textrm{c} + i\eta _\textrm{c}\gamma _\textrm{w}d_\textrm{w, SSC}^\textrm{max}/\eta _\textrm{w} \end{pmatrix}. \end{aligned}$$

Here, we ignore the product of $d^\textrm{max}_\textrm{w, SSC}$ and $\Delta \phi _\textrm{c}$ in this calculation, as it is sufficiently small. By substituting Eq. (12) into Eqs. (2) and (3), the absorptance $A'_\textrm{SSC}$ is given by

13 $$\begin{aligned} A'_\textrm{SSC} = \frac{-4\textrm{Im}(\varepsilon _\textrm{w})/|\varepsilon _\textrm{w}|}{(1 - \textrm{Im}(\varepsilon _\textrm{w})/|\varepsilon _\textrm{w}|)^2 + (\textrm{Re}(\varepsilon _\textrm{w})/|\varepsilon _\textrm{w}| - n_\textrm{c}^2\textrm{Im}(n_\textrm{m})/|n_\textrm{m}|^2n_\textrm{i} + \Delta \phi _\textrm{c}n_\textrm{c}/n_\textrm{i})^2}. \end{aligned}$$

Here, we ignore the product of $\textrm{Im}(\eta _\textrm{m})\cdot d^\textrm{max}_\textrm{w, SSC}$ and $\textrm{Im}(\eta _\textrm{m})\cdot \Delta \phi _\textrm{c}$ because the imaginary part of the refractive index of the metallic mirror is large, implying that the imaginary part of the impedance of the metallic mirror is small. The thickness of the dielectric layer at the maximum absorptance, $d_\textrm{c, SSC}^\textrm{max}$, and the maximum absorptance $A_\textrm{SSC}^{'\mathrm{max}}$ are given by

14 $$\begin{aligned} d_\textrm{c, SSC}^\textrm{max} = \frac{\lambda _0}{4n_\textrm{c}} - \frac{{n_\textrm{i}\mathrm{Re}}(\varepsilon _\textrm{w})}{k_0n_\textrm{c}^2|\varepsilon _\textrm{w}|} + \frac{\textrm{Im}(n_\textrm{m})}{k_0|n_\textrm{m}|^2} \end{aligned}$$

and

15 $$\begin{aligned} A_\textrm{SSC}^{'\textrm{max}} = \frac{-4\textrm{Im}(\varepsilon _\textrm{w})}{|\varepsilon _\textrm{w}|(1 - \textrm{Im}(\varepsilon _\textrm{w})/|\varepsilon _\textrm{w}|)^2}. \end{aligned}$$

As a result, it was found that the thickness of the dielectric layer at the maximum absorptance was less than one-quarter of the incident photon wavelength scaled by its refractive index. This is because of the real part of the relative permittivity of the wire layer and imaginary part of the refractive index of the metal.

### Double-side optical cavity

As shown in Fig. 1b, we apply the transmission line model to SSPDs with double-side optical cavities. These SSPDs have dielectric layers placed both above and below the superconducting wire, and a metallic mirror is placed on top of the upper dielectric layer. First, to derive the thickness of the wire layer to achieve the maximum absorptance, we used a PEC mirror as the metallic mirror and set the thicknesses of the upper and lower dielectric layers as one-quarter of the incident photon wavelength scaled by their refractive indices. The *F* matrix of the SSPDs with double-side optical cavities is simplified as

16 $$\begin{aligned} F_\textrm{DSC} = F_\textrm{c1}\times F_\textrm{w}\times F_\textrm{c2} = \begin{pmatrix} -\eta _\textrm{c1}/\eta _\textrm{c2}& -\eta _\textrm{c1}\eta _\textrm{c2}\gamma _\textrm{w}d_\textrm{w}/\eta _\textrm{w}\\ -\eta _\textrm{w}\gamma _\textrm{w}d_\textrm{w}/\eta _\textrm{c1}\eta _\textrm{c2}& -\eta _\textrm{c2}/\eta _\textrm{c1} \end{pmatrix}, \end{aligned}$$

where the subscripts $\textrm{c1}$ and $\textrm{c2}$ denote the lower and upper dielectric layers, respectively, as shown in Fig. 1b. We used a similar approach for SSPDs with single-side optical cavities to derive the dependence of the absorptance ($A_\textrm{DSC}$) on the thickness of the wire layer, thickness of the wire layer at the maximum absorptance $d_\textrm{w, DSC}^\textrm{max}$, and the maximum absorptance $A_\textrm{DSC}^\textrm{max}$. $A_\textrm{DSC}$, $d_\textrm{w, DSC}^\textrm{max}$, and $A_\textrm{DSC}^\textrm{max}$ are given by

17 $$\begin{aligned} A_\textrm{DSC} = \frac{-4k_0n_\textrm{i}d_\textrm{w}\textrm{Im}(\varepsilon _\textrm{w})/n_\textrm{c1}^2}{(k_0n_\textrm{i}d_\textrm{w}\textrm{Im}(\varepsilon _\textrm{w})/n_\textrm{c1}^2 - 1)^2 +~(k_0n_\textrm{i}d_\textrm{w}\textrm{Re}(\varepsilon _\textrm{w})/n_\textrm{c1}^2)^2}, \end{aligned}$$

18 $$\begin{aligned} d_\textrm{w, DSC}^\textrm{max} = \frac{n_\textrm{c1}^2}{k_0n_\textrm{i}|\varepsilon _\textrm{w}|}, \end{aligned}$$

and

19 $$\begin{aligned} A_\textrm{DSC}^\textrm{max} = \frac{2\textrm{Im}(\varepsilon _\textrm{w})}{\textrm{Im}(\varepsilon _\textrm{w}) - |\varepsilon _\textrm{w}|}. \end{aligned}$$

The results indicate that $d_\textrm{w, DSC}^\textrm{max}$ depends on not only the refractive index of the input medium and complex relative permittivity of the wire layer but also the refractive index of the lower dielectric layer. The maximum absorptance of the SSPDs with double-side optical cavities, $A_\textrm{DSC}^\textrm{max}$, is the same as that of SSPDs with single-side optical cavities, as shown in Eq. (11).

Next, to derive the thickness of the lower and upper dielectric layers to achieve the maximum absorptance, we used the thickness of the wire layer given by Eq. (18) and applied the characteristic of the metallic mirror, i.e., $\eta _\textrm{m}\ne 0$. The *F* matrix of SSPDs with double-side optical cavities is given by

20 $$\begin{aligned} F'_\textrm{DSC} = \begin{pmatrix} -\frac{\eta _\textrm{c1}}{\eta _\textrm{c2}}& -i(\eta _\textrm{c2}\Delta \phi _\textrm{c1} + \eta _\textrm{c1}\Delta \phi _\textrm{c2}) - \frac{\eta _\textrm{c1}\eta _\textrm{c2}\gamma _\textrm{w}d_\textrm{w}}{\eta _\textrm{w}}\\ -i\left( \frac{\Delta \phi _\textrm{c2}}{\eta _\textrm{c1}} + \frac{\Delta \phi _\textrm{c1}}{\eta _\textrm{c2}}\right) - \frac{\eta _\textrm{w}\gamma _\textrm{w}d_\textrm{w}}{\eta _\textrm{c1}\eta _\textrm{c2}}& -\frac{\eta _\textrm{c2}}{\eta _\textrm{c1}} \end{pmatrix}, \end{aligned}$$

where $\Delta \phi _\textrm{c1}$ ($\Delta \phi _\textrm{c2}$) is defined by $\phi _\textrm{c1} = \pi /2 + \Delta \phi _\textrm{c1}$ ($\phi _\textrm{c2} = \pi /2 + \Delta \phi _\textrm{c2}$), where $\phi _\textrm{c1}=k_0n_\textrm{c1}d_\textrm{c1}$ ($\phi _\textrm{c2}=k_0n_\textrm{c2}d_\textrm{c2}$), and $n_\textrm{c1}$ ($n_\textrm{c2}$) and $d_\textrm{c1}$ ($d_\textrm{c2}$) are the refractive index and thickness of the lower (upper) dielectric layer, respectvely. Here, we ignore the products of $\Delta \phi _\textrm{c1}$, $\Delta \phi _\textrm{c2}$, and $d_\textrm{w}$, as their values are small. As in the derivation of the equations for SSPDs with single-side optical cavities, the absorptance $A'_\textrm{DSC}$ is given by

21 $$\begin{aligned} A'_\textrm{DSC} = \frac{-4\textrm{Im}(\varepsilon _\textrm{w})/|\varepsilon _\textrm{w}|}{(1 - \textrm{Im}(\varepsilon _\textrm{w})/|\varepsilon _\textrm{w}|)^2 +~(\textrm{Re}(\varepsilon _\textrm{w})/|\varepsilon _\textrm{w}| + \Delta \phi _\textrm{DSC}n_\textrm{i}/n_\textrm{c1} - n^2_\textrm{c2}n_\textrm{i}\textrm{Im}(n_\textrm{m})/n_\textrm{c1}^2|n_\textrm{m}|^2)^2}, \end{aligned}$$

where $\Delta \phi _\textrm{DSC}$ is defined by

22 $$\begin{aligned} \Delta \phi _\textrm{DSC} = \Delta \phi _\textrm{c1} + \frac{n_\textrm{c2}}{n_\textrm{c1}}\Delta \phi _\textrm{c2}. \end{aligned}$$

Therefore, $\Delta \phi _\textrm{DSC}^\textrm{max}$, which is the value of $\Delta \phi _\textrm{DSC}$ at the maximum absorptance, and the maximum absorptance $A_\textrm{DSC}^{'\mathrm{max}}$ are given by

23 $$\begin{aligned} \Delta \phi _\textrm{DSC}^\textrm{max} = -\frac{n_\textrm{c1}}{n_\textrm{i}}\frac{\textrm{Re}(\varepsilon _\textrm{w})}{|\varepsilon _\textrm{w}|} + \frac{n_\textrm{c2}^2\textrm{Im}(n_\textrm{m})}{n_\textrm{c1}|n_\textrm{m}|^2} \end{aligned}$$

and

24 $$\begin{aligned} A_\textrm{DSC}^{'\mathrm{max}} = \frac{-4\textrm{Im}(\varepsilon _\textrm{w})}{|\varepsilon _\textrm{w}|(1 - \textrm{Im}(\varepsilon _\textrm{w})/|\varepsilon _\textrm{w}|)^2}. \end{aligned}$$

The results indicate that the maximum absorptance of SSPDs with double-side optical cavities is the same as that of single-side optical cavities, as shown in Eq. (15). As shown in Eqs. (22) and  (23), various combinations of the thicknesses of the lower and upper dielectric layers can yield the maximum absorptance, as $\Delta \phi _\textrm{c1}$ and $\Delta \phi _\textrm{c2}$ depend on the thickness of the upper and lower dielectric layers, respectively.

We present an example of the thickness combinations of the two dielectric layers that achieve the maximum absorptance. When the thickness of the lower dielectric layer is one-quarter of the incident photon wavelength scaled by its refractive index, i.e., $d_\textrm{c1} = \lambda _0/4n_\textrm{c1}$, the thickness of the upper dielectric layer that yields the maximum absorptance, $d_\textrm{c2, DSC}^\textrm{max}$, is given by

25 $$\begin{aligned} d_\textrm{c2, DSC}^\textrm{max} = \frac{\lambda _0}{4n_\textrm{c2}} - \frac{n_\textrm{c1}^2\textrm{Re}(\varepsilon _\textrm{w})}{k_0n_\textrm{i}n_\textrm{c2}^2|\varepsilon _\textrm{w}|} + \frac{\textrm{Im}(n_\textrm{m})}{k_0|n_\textrm{m}|^2}. \end{aligned}$$

The result indicates that the thickness of the dielectric layer at the maximum absorptance is less than one-quarter of the incident photon wavelength scaled by its refractive index. This is because of the real part of the relative permittivity of the wire layer and imaginary part of the refractive index of the metal.

### Dielectric multi-layer optical cavity

Here, we apply the transmission line model to SSPDs with dielectric multi-layer optical cavities, shown in Fig. 1c. The dielectric multi-layer optical cavities comprise two dielectric layers with different refractive indices, which are alternatively stacked on the superconducting wire. The thickness of each layer is one-quarter of the incident photon wavelength scaled by its refractive index.

The *F* matrix of two dielectric layers, indicated by subscripts $\textrm{c1}$ and $\textrm{c2}$, are given by

26 $$\begin{aligned} F_\textrm{c1} = \begin{pmatrix} 0& i\eta _\textrm{c1}\\ i/\eta _\textrm{c1}& 0 \end{pmatrix} \end{aligned}$$

and

27 $$\begin{aligned} F_\textrm{c2} = \begin{pmatrix} 0& i\eta _\textrm{c2}\\ i/\eta _\textrm{c2}& 0 \end{pmatrix}. \end{aligned}$$

The *F* matrix of the two stacked dielectric layers with different refractive indices (one period), $F_\textrm{c12}$, is given by

28 $$\begin{aligned} F_\textrm{c12} = F_\textrm{c1}F_\textrm{c2} = \begin{pmatrix} -\eta _\textrm{c1}/\eta _\textrm{c2}& 0\\ 0& -\eta _\textrm{c2}/\eta _\textrm{c1} \end{pmatrix}. \end{aligned}$$

Therefore, the *F* matrix for *N* periods is given by

29 $$\begin{aligned} F_\textrm{c12}^N = \begin{pmatrix} ~(-\eta _\textrm{c1}/\eta _\textrm{c2})^N& 0\\ 0& (-\eta _\textrm{c2}/\eta _\textrm{c1})^N \end{pmatrix}. \end{aligned}$$

The *F* matrix of SSPDs with dielectric multi-layer optical cavities, $F_\textrm{MLC}$, is given by

30 $$\begin{aligned} F_\textrm{MLC} = F_\textrm{w}F_\textrm{c12}^N = \begin{pmatrix} ~(-\eta _\textrm{c1}/\eta _\textrm{c2})^N& (-\eta _\textrm{c2}/\eta _\textrm{c1})^N\eta _\textrm{w}\gamma _\textrm{w}d_\textrm{w}\\ ~(-\eta _\textrm{c1}/\eta _\textrm{c2})^N\gamma _\textrm{w}d_\textrm{w}/\eta _\textrm{w}& (-\eta _\textrm{c2}/\eta _\textrm{c1})^N \end{pmatrix}. \end{aligned}$$

Substituting Eq. (30) into Eqs. (3) and  (4), the reflection coefficient $r_\textrm{MLC}$ and transmission coefficient $t_\textrm{MLC}$ of SSPDs with dielectric multi-layer optical cavities are given by

31 $$\begin{aligned} r_\textrm{MLC} = \frac{(-\eta _\textrm{c1}/\eta _\textrm{c2})^N\eta _\textrm{o} +~(-\eta _\textrm{c2}/\eta _\textrm{c1})^N\eta _\textrm{w}\gamma _\textrm{w}d_\textrm{w} -~(-\eta _\textrm{c1}/\eta _\textrm{c2})^N\gamma _\textrm{w}\eta _\textrm{o}\eta _\textrm{i}/\eta _\textrm{w} -~(-\eta _\textrm{c2}/\eta _\textrm{c1})^N\eta _\textrm{i}}{(-\eta _\textrm{c1}/\eta _\textrm{c2})^N\eta _\textrm{o} +~(-\eta _\textrm{c2}/\eta _\textrm{c1})^N\eta _\textrm{w}\gamma _\textrm{w}d_\textrm{w} +~(-\eta _\textrm{c1}/\eta _\textrm{c2})^N\gamma _\textrm{w}\eta _\textrm{o}\eta _\textrm{i}/\eta _\textrm{w} +~(-\eta _\textrm{c2}/\eta _\textrm{c1})^N\eta _\textrm{i}} \end{aligned}$$

and

32 $$\begin{aligned} t_\textrm{MLC} = \frac{2\eta _\textrm{o}\eta _\textrm{i}}{(-\eta _\textrm{c1}/\eta _\textrm{c2})^N\eta _\textrm{o} +~(-\eta _\textrm{c2}/\eta _\textrm{c1})^N\eta _\textrm{w}\gamma _\textrm{w}d_\textrm{w} +~(-\eta _\textrm{c1}/\eta _\textrm{c2})^N\gamma _\textrm{w}\eta _\textrm{o}\eta _\textrm{i}/\eta _\textrm{w} +~(-\eta _\textrm{c2}/\eta _\textrm{c1})^N\eta _\textrm{i}}. \end{aligned}$$

Here, we ignore the imaginary part of the refractive indices of the input and output media. By assuming that the refractive indices satisfy $n_\textrm{c1} < n_\textrm{c2}$, resulting in $\eta _\textrm{c1} > \eta _\textrm{c2}$, and *N* is sufficiently large, i.e., $|-\eta _\textrm{c1}/\eta _\textrm{c2}|^N \gg |-\eta _\textrm{c2}/\eta _\textrm{c1}|^N$, the transmission coefficient becomes negligible. The reflection coefficient can be approximated by

33 $$\begin{aligned} r_\textrm{MLC} = \frac{1 - \gamma _\textrm{w}d_\textrm{w}\eta _\textrm{i}/\eta _\textrm{w}}{1 + \gamma _\textrm{w}d_\textrm{w}\eta _\textrm{i}/\eta _\textrm{w}}. \end{aligned}$$

By substituting Eq. (33) into Eq. (2), the absorptance of SSPDs with dielectric multi-layer optical cavities is given by

34 $$\begin{aligned} A_\textrm{MLC} = \frac{-4k_0\textrm{Im}(\varepsilon _\textrm{w})n_\textrm{i}d_\textrm{w}}{(n_\textrm{i} - k_0\textrm{Im}(\varepsilon _\textrm{w})d_\textrm{w})^2 +~(k_0\textrm{Re}(\varepsilon _\textrm{w})d_\textrm{w})^2}. \end{aligned}$$

The result is the same as the absorptance of SSPDs with single-side optical cavities, as shown in Eq. (9). It indicates that the thickness of the wire layer at the maximum absorptance, $d_\textrm{MLC}^\textrm{max}$, and the maximum absorptance $A_\textrm{MLC}^\textrm{max}$ are the same as those of SSPDs with single-side optical cavities given by

35 $$\begin{aligned} d^\textrm{max}_\textrm{MLC} = \frac{n_\textrm{i}}{k_0|\varepsilon _\textrm{w}|} \end{aligned}$$

and

36 $$\begin{aligned} A_\textrm{MLC}^\textrm{max} = \frac{2\textrm{Im}(\varepsilon _\textrm{w})}{\textrm{Im}(\varepsilon _\textrm{w}) - |\varepsilon _\textrm{w}|}. \end{aligned}$$

It should be noted that from Eqs. (11),  (19), and  (36), the maximum absorptance of SSPDs with single-side, double-side, and dielectric multi-layer optical cavities is described by the same formula. From Eqs. (10) and  (35), the thickness of the wire layer at the maximum absorptance is described by the same expression for both the single-side optical cavity and the dielectric multi-layer optical cavity. In these cases, it depends on the refractive index of the input media. In contrast, Eq. (18) indicates that, in the double-side optical cavity, the thickness of the wire layer at the maximum absorptance can be tuned through the combination of the refractive index of the input medium and the lower dielectric layer.

## Comparisons with numerical simulations

**Table 1:** Materials and refractive indices used for the analytical formulae and simulations. Note that the refractive index of the PEC mirror was set to $-1000i$ in the RCWA and FEM simulations, as it was not possible to set an infinite refractive index.

Material	Refractive index	Reference
Vacuum	1	
NbN	$4.905 - 4.293i$	^36^
Si	3.628	^36^
SiO	1.551	^36^
SiO$_2$	1.444	^36^
Ta$_2$O$_5$	2.15	^7^
PEC	$-1000i$	
Ag	$0.322 - 10.99i$	^36^

**Fig. 2:**
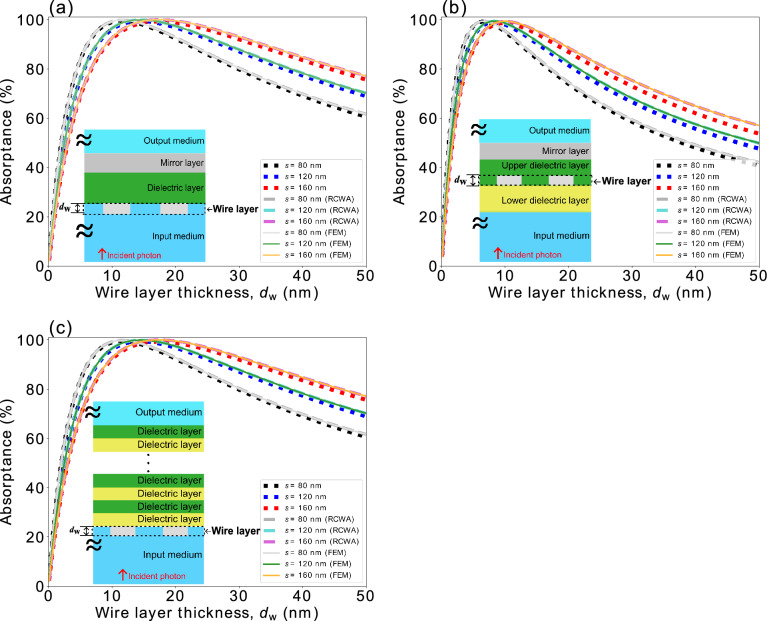
Comparisons of analytical and simulation results. In each figure, the results represented by the dotted lines are calculated using the analytical formulae given by the transmission line model, and those represented by dashed (solid) lines are obtained from the simulations using RCWA (FEM). (**a**) Dependence of the absorptance on the thickness of the wire layer of SSPDs with single-side optical cavities. (**b**)  Dependence of the absorptance on the thickness of the wire layer of SSPDs with double-side optical cavities. (**c**) Dependence of the absorptance on the thickness of the wire layer of SSPDs with dielectric multi-layer optical cavities.

**Fig. 3:**
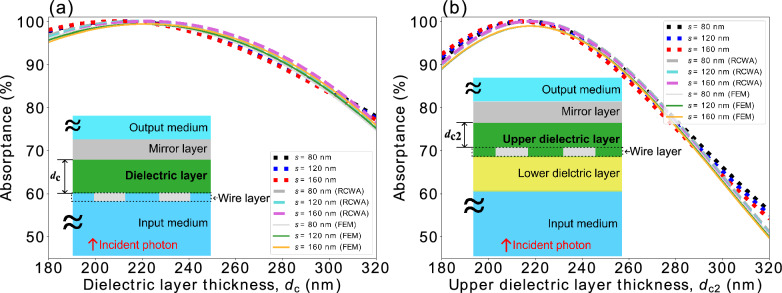
Comparisons between analytical and simulation results. In each figure, the results represented by the dotted lines are calculated using the analytical formula given by the transmission line model, and those represented by the dashed (solid) lines are obtained from simulations using RCWA (FEM). (**a**) Dependence of the absorptance on the thickness of the dielectric layer of SSPDs with single-side optical cavities. (**b**) Dependence of the absorptance on the thickness of the upper dielectric layer of SSPDs with double-side optical cavities.

Next, we compared the results obtained from the transmission line model with those from the simulation methods, RCWA and FEM. $S^4$ (Stanford Stratified Structure Solver)^45^ was used for the RCWA simulations, and COMSOL Multiphysics with RF module was used for the FEM simulations. The derivation of the absorptance using COMSOL Multiphysics with RF module follows the method described in^35^. In these comparisons, the wavelength of the incident photon was set to $1550~\textrm{nm}$. We used the refractive indices of the superconducting material, dielectric layer, metallic mirror in the optical cavities, and input medium reported in^7,36^ and summarized in Table 1. Note that the refractive index of the PEC mirror was set to $-1000i$ in the RCWA and FEM simulations, as it was not possible to set an infinite refractive index. In the comparisons, NbN was used as the superconducting wire, and the line width was fixed at $80~\textrm{nm}$. The slit widths (filling factors) were set to $s = 80~\textrm{nm}$ ($f = 0.50$), $s = 120~\textrm{nm}$ ($f = 0.40$), and $s = 160~\textrm{nm}$ ($f = 0.33$), satisfying Condition I. The thickness of the PEC or metallic mirror in SSPDs with single-side and double-side optical cavities in the simulation was fixed at $130~\textrm{nm}$, which satisfied Condition VI. SSPDs with single-side and dielectric multi-layer optical cavities used vacuum as both the input and output media. In SSPDs with single-side optical cavities, the dielectric layers were made of SiO, whereas SiO$_2$ and Ta$_2$O$_5$ were used in dielectric multi-layer optical cavities. In the configuration of SSPDs with double-side optical cavities, Si was used as the input medium because the model assumes backside illumination through a Si substrate, which is commonly employed in practical device structure^32^, while the output medium was assumed to be vacuum. In this case, when the Si substrate is sufficiently thick and an appropriate anti-reflection coating is formed between the vacuum and Si substrate, the incident light can be regarded as entering from Si, so that Si can be treated as the input medium. The lower and upper dielectric layers were assumed to be SiO$_2$ and SiO, respectively.

First, we compared the dependences of the absorptance on the thickness of the wire layer given by the analytical formulae for SSPDs with single-side optical cavities (Eq. 9), double-side optical cavities (Eq. 17), and dielectric multi-layer optical cavities (Eq. 34) with the simulation results. In these calculations and simulations, we fixed the thickness of the dielectric layer at one-quarter of the incident photon wavelength scaled by its refractive index. Figure 2a–c show the results for SSPDs with single-side, double-side, and dielectric multi-layer optical cavities, respectively. In each figure, the dotted lines show the results given by the analytical formulae, and dashed and solid lines indicate the RCWA and FEM simulation results, respectively. The analytical and simulation results are nearly identical. Table 2 summarizes the thicknesses of the wire layer that achieve the maximum absorptance in SSPDs with single-side optical cavities (Eq. 10), and double-side optical cavities (Eq. 18), and dielectric multi-layer optical cavities (Eq. 35) and the corresponding results of RCWA and FEM. The thickness of the wire layer that achieves the maximum absorptance differs by less than 2% between the analytical results and the simulation results. These results indicate that the analytical formulae can reliably determine the thickness of the wire layer required to achieve the maximum absorptance. The slight difference between the analytical and numerical results at larger wire thickness arises from the approximations introduced in Condition VII, which falls outside the range of validity in this regime.

Next, we compared the dependence of the absorptance on the thickness of the dielectric layer given by the analytical formula for SSPDs with single-side optical cavities (Eq. 13) and double-side optical cavities (Eq. 21) with that shown in the simulation results. We assumed the material of the metallic mirror as Ag in both the optical cavities. In the SSPDs with double-side optical cavities, the thickness of the lower dielectric layer was fixed at the quarter-wave thickness of the incident photon wavelength scaled by its refractive index. As shown in Fig. 3, the analytical and simulation results are in good agreement. Table 2 summarizes the thickness of the dielectric layer that achieves the maximum absorptance in SSPDs with single-side optical cavities (Eq. 14) and double-side optical cavities (Eq. 25). The difference between the thickness of the dielectric layer to achieve maximum absorptance given by the analytical formulae and that given by simulations is less than 6%. Thus, the analytical and simulation results are nearly identical, confirming that the analytical approach is useful in obtaining the dielectric layer thickness at the maximum absorptance. The slight discrepancy between analytical and simulation results increases as the thickness of dielectric layer deviates from quarter-wave thickness of the incident photon wavelength scaled by its refractive index, because the approximation introduced in Eq. (7) falls outside its range of validity in this regime. The source codes used to reproduce these results are provided in^46^.

**Table 2:** Thickness of the wire layer or dielectric layer required to achieve the maximum absorptance given by the analytical formulae and by simulations using RCWA and FEM.

Single-side optical cavity							
$d^\textrm{max}_\textrm{w, SSC}$				$d^\textrm{max}_\textrm{c, SSC}$			
Slit width	Eq. (10)	RCWA	FEM	Slit width	Eq. (14)	RCWA	FEM
$80~\mathrm{\upmu m}$	$11.6~\textrm{nm}$	$11.6~\textrm{nm}$	$11.6~\textrm{nm}$	$80~\mathrm{\upmu m}$	$211~\textrm{nm}$	$217~\textrm{nm}$	$217~\textrm{nm}$
$120~\mathrm{\upmu m}$	$14.4~\textrm{nm}$	$14.5~\textrm{nm}$	$14.5~\textrm{nm}$	$120~\mathrm{\upmu m}$	$210~\textrm{nm}$	$219~\textrm{nm}$	$219~\textrm{nm}$
$160~\mathrm{\upmu m}$	$17.3~\textrm{nm}$	$17.4~\textrm{nm}$	$17.4~\textrm{nm}$	$160~\mathrm{\upmu m}$	$209~\textrm{nm}$	$222~\textrm{nm}$	$222~\textrm{nm}$

Double-side optical cavity							
$d^\textrm{max}_\textrm{w, DSC}$				$d^\textrm{max}_\textrm{c2, DSC}$			
Slit width	Eq. (18)	RCWA	FEM	Slit width	Eq. (25)	RCWA	FEM
$80~\mathrm{\upmu m}$	$6.6~\textrm{nm}$	$6.6~\textrm{nm}$	$6.6~\textrm{nm}$	$80~\mathrm{\upmu m}$	$216~\textrm{nm}$	$218~\textrm{nm}$	$218~\textrm{nm}$
$120~\mathrm{\upmu m}$	$8.2~\textrm{nm}$	$8.3~\textrm{nm}$	$8.3~\textrm{nm}$	$120~\mathrm{\upmu m}$	$215~\textrm{nm}$	$218~\textrm{nm}$	$218~\textrm{nm}$
$160~\mathrm{\upmu m}$	$9.8~\textrm{nm}$	$10.0~\textrm{nm}$	$10.0~\textrm{nm}$	$160~\mathrm{\upmu m}$	$213~\textrm{nm}$	$217~\textrm{nm}$	$217~\textrm{nm}$

Dielectric multi-layer optical cavity							
$$d^\textrm{max}_\textrm{w, MLC}$$							
Slit width	Eq. (35)	RCWA	FEM				
$80~\mathrm{\upmu m}$	$11.6~\textrm{nm}$	$11.6~\textrm{nm}$	$11.6~\textrm{nm}$				
$120~\mathrm{\upmu m}$	$14.4~\textrm{nm}$	$14.5~\textrm{nm}$	$14.5~\textrm{nm}$				
$160~\mathrm{\upmu m}$	$17.3~\textrm{nm}$	$17.4~\textrm{nm}$	$17.4~\textrm{nm}$				

## Discussion

### Physical mechanism to achieve maximum absorptance

In this section, by introducing the impedance model, we clarify the physical mechanism by which the geometry of SSPDs with optical cavities achieves the maximum absorptance and show that it can be interpreted as an impedance matching condition between the SSPDs with optical cavity and the input medium. The *F* matrix is transformed into the input impedance $\eta _\textrm{in}$, which is expressed as^41^

37 $$\begin{aligned} \eta _\textrm{in} = \frac{F_{11}\eta _\textrm{o} + F_{12}}{F_{21}\eta _\textrm{o} + F_{22}}. \end{aligned}$$

By substituting Eqs. (8),  (16), and  (30) into Eq. (37), the input impedances of SSPDs with single-side optical cavities ($\eta _\textrm{in, SSC}$), double-side optical cavities ($\eta _\textrm{in, DSC}$), and dielectric multi-layer optical cavities ($\eta _\textrm{in, MLC}$) are expressed as

38 $$\begin{aligned} \eta _\textrm{in, SSC} = \frac{\eta _0}{ik_0\varepsilon _\textrm{w}d_\textrm{w}}, \end{aligned}$$

39 $$\begin{aligned} \eta _\textrm{in, DSC} = \frac{i\eta _0k_0\varepsilon _\textrm{w}d_\textrm{w}}{n_\textrm{c1}^2}, \end{aligned}$$

and

40 $$\begin{aligned} \eta _\textrm{in, MLC} = \frac{\eta _0}{ik_0\varepsilon _\textrm{w}d_\textrm{w}}. \end{aligned}$$

Here, we assume the PEC mirror, i.e., $\eta _\textrm{o} = 0$, in SSPDs with single-side and double-side optical cavities. For SSPDs with dielectric multi-layer optical cavities, the number of periods is sufficiently large. By substituting Eq. (10) into Eq. (38), Eq. (18) into Eq. (39), and Eq. (35) into Eq. (40), the input impedance of SSPDs with single-side optical cavities ($\eta ^\textrm{max}_\textrm{in, SSC}$), double-side optical cavities ($\eta ^\textrm{max}_\textrm{in, DSC}$), and dielectric multi-layer optical cavities ($\eta ^\textrm{max}_\textrm{in, MLC}$), required to achieve the maximum absorptance, satisfies the following equations:

41 $$\begin{aligned} \eta ^\textrm{max}_\textrm{in, SSC} = \frac{\eta _\textrm{i}|\varepsilon _\textrm{w}|}{i\varepsilon _\textrm{w}}, \end{aligned}$$

42 $$\begin{aligned} \eta ^\textrm{max}_\textrm{in, DSC} = \frac{i\eta _\textrm{i}\varepsilon _\textrm{w}}{|\varepsilon _\textrm{w}|}, \end{aligned}$$

and

43 $$\begin{aligned} \eta ^\textrm{max}_\textrm{in, MLC} = \frac{\eta _\textrm{i}|\varepsilon _\textrm{w}|}{i\varepsilon _\textrm{w}}. \end{aligned}$$

The results indicate that the absolute values of the input impedance for SSPDs with single-side, double-side, and dielectric multi-layer optical cavities should be identical to the input impedance, i.e., $|\eta _\textrm{in}|=\eta _\textrm{i}$, for achieving the maximum absorptance. Therefore, the maximum absorptance is achieved when the impedance of the input medium matches the input impedance of SSPDs with optical cavities. In other words, the reflectance becomes minimum.

**Fig. 4:**
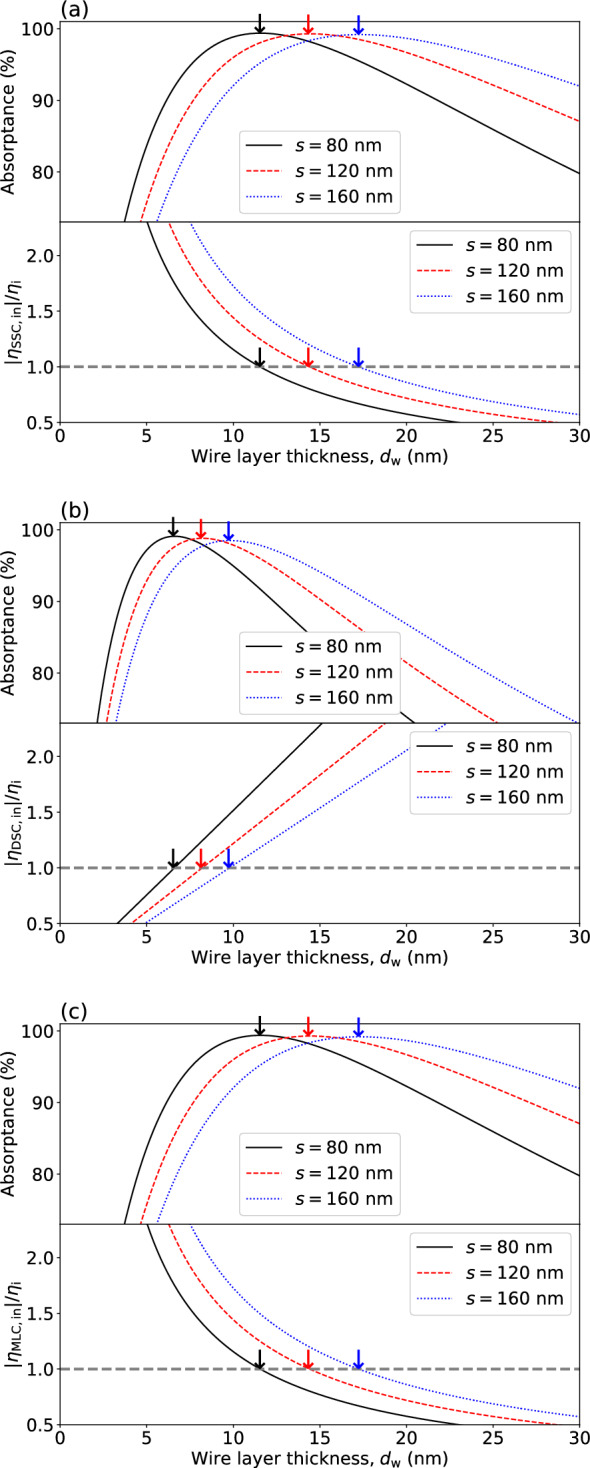
Dependence of the absorptance and ratio of the input impedance to the impedance of the input media on the thickness of the wire layer. The upper panel of each figure shows the absorptance as a function of the thickness of the wire layer, determined by Eq. (9) in (**a**), Eq. (17) in (**b**), and Eq. (34) in (**c**). The lower panel shows the ratio of the absolute value of the input impedance of SSPDs with the cavities to the impedance of the input medium. (**a**) SSPDs with single-side optical cavities. (**b**) SSPDs with double-side optical cavities. (**c**) SSPDs with dielectric multi-layer optical cavities. The arrows indicate the maximum absorptance and corresponding ratio of the input impedance to the impedance of the input media.

Figure 4 shows the dependence of the absorptance and ratio of input impedance to impedance of the input medium on the thickness of SSPDs with optical cavities. In this calculation, the geometry of SSPDs with optical cavities is the same as that used in the previous section. At the maximum absorptance, the absolute value of the input impedance of SSPDs with optical cavities $|\eta _\textrm{in}|$ approaches the impedance of the input media $\eta _\textrm{i}$, i.e., impedance matching is achieved. The source codes used to reproduce these results are provided in^46^.

### Physical role of lower dielectric layer in double-side optical cavity

We investigated the physical role of the lower dielectric layer by applying the quarter-wave impedance transformer^41^ to SSPDs with double-side optical cavities. In SSPDs with double-side optical cavities, the input impedance from the lower dielectric layer, $\eta '_\textrm{in, DSC}$, is given by

44 $$\begin{aligned} \eta '_\textrm{in, DSC} = \frac{\eta _0}{ik_0\varepsilon _\textrm{w}d_\textrm{w}} \end{aligned}$$

in the case where the metallic mirror is assumed to be a PEC mirror. The quarter-wave transformer has the characteristic impedance that satisfies $\eta _\textrm{QWT} = \sqrt{\eta _\textrm{i}\eta _\textrm{o}}$, where $\eta _\textrm{i}$ ($\eta _\textrm{o}$) represents the impedance of the input (output) medium. In SSPDs with double-side optical cavities, by replacing $\eta _\textrm{o}$ with $\eta '_\textrm{in, DSC}$, a $\eta _\textrm{QWT}$ is given by

45 $$\begin{aligned} \eta _\textrm{QWT} = \sqrt{\frac{\eta _0\eta _\textrm{i}}{k_0\varepsilon _\textrm{w}d_\textrm{w}}}. \end{aligned}$$

From Eq. (45), we can derive

46 $$\begin{aligned} d_\textrm{w} = \frac{n_\textrm{QWT}^2}{k_0n_\textrm{i}|\varepsilon _\textrm{w}|}. \end{aligned}$$

The result indicates that the lower dielectric layer in the double-side optical cavities works as a quarter-wave impedance transformer, as Eq. (46) is identical to Eq. (18) when $n_\textrm{QWT}\rightarrow n_\textrm{c1}$. Therefore, the absorptance of SSPDs with double-side optical cavities depends on the refractive index of the lower dielectric layer. In other words, the refractive index of the lower dielectric layer needs to be equal to Eq. (45), i.e., $\eta _\textrm{c1} = \eta _\textrm{QWT}$, to achieve the maximum absorptance.

## Device design guidelines for SSPDs with optical cavities

In this section, we summarize a practical design flow for SSPDs with optical cavities based on the analytical results obtained from transmission line and impedance models. The first step is to define the target wavelength at which high absorptance is required. Next, the cavity type, superconducting material, dielectric material, mirror material, and filling factor are selected according to the application requirements and fabrication constraints. In general, different cavity types involve different design characteristics and trade-offs. For example, the single-side optical cavity has a simple structure and is easy to fabricate, whereas the double-side optical cavity provides additional design flexibility through the combination of the input medium and the lower dielectric layer. The dielectric multi-layer optical cavity can achieve high absorptance over a broad bandwidth for suitable combinations of the two dielectric refractive indices, although it requires a more complex multi-layer fabrication process. Note that, in SSPDs with double-side optical cavities, the refractive index of the lower dielectric layer should be chosen to satisfy the role of quarter-wave impedance transformer to achieve the maximum absorptance. In SSPDs with dielectric multi-layer optical cavities, the layers are stacked such that the dielectric layer adjacent to the superconducting wire has a smaller refractive index than the next layer, i.e. $n_\textrm{c1} < n_\textrm{c2}$ in Fig. 1c. The thickness of wire layer are chosen to satisfy Eq. (10) in SSPDs with single-side optical cavities, Eq. (18) in SSPDs with double-side optical cavities, and Eq. (35) in SSPDs with dielectric multi-layer optical cavities. Similarly, the thickness of dielectric layer are chosen to satisfy Eq. (14) in SSPDs with single-side optical cavities, and Eq. (23) in SSPDs with double-side optical cavities. Finally, using the impedance model given by Eq. (37), one can check that the input impedance of the SSPDs with optical cavities ($\eta _\textrm{in}$) is close to the impedance of the input medium ($\eta _\textrm{i}$), which ensures the maximum absorptance.

## Conclusions

We derived the analytical formulae for the absorptance of SSPDs with optical cavities, the maximum absorptance, and required thickness of the wire layer and dielectric layer to achieve the maximum absorptance by applying the transmission line model. We compared the results given by the analytical formulae proposed in this study with those obtained from simulations conducted using RCWA and FEM. These comparisons showed that the results are nearly identical. We investigated the physical mechanism underlying the maximum absorptance of SSPDs with optical cavities by introducing the impedance model. The maximum absorptance of SSPDs with optical cavities is achieved when the impedance of the input medium and input impedance of SSPDs with optical cavities match. We clarified that the lower dielectric layer in the double-side optical cavities works as a quarter-wave impedance transformer, and reported the refractive index of the lower dielectric layer required to achieve the maximum absorptance. The design concepts proposed in this study are applicable to other superconducting detectors with optical cavities, such as MKIDs^47^ and transition-edge sensors (TESs)^48^.

## Data Availability

The source code to reproduce the figures and table is available in Zenodo with the identifier DOI: https://doi.org/10.5281/zenodo.19722815.
